# Styptic Collodion Formula

**Published:** 1874-01

**Authors:** 


Styptic Collodion.—The following will be found a most useful formula:

| | |
|---|---|
| ℞.—Tannin, . . . . . | 2 ozs.; |
| Alcohol, . . . . . | 4 ozs., fl.; |
| Ether, . . . . . . | 12 ozs., fl.; |
| Soluble Cotton, . . . . | 1 dr. and 2 scruples; |
| Canada Balsam, . . . | 1 drachm. |

Dissolve the tannin in one part of a mixture of alcohol and ether, with the Canada balsam; then add the cotton.—*Dublin Medical Press and Circular.*

